# Deciphering Genotype-by- Environment Interaction for Targeting Test Environments and Rust Resistant Genotypes in Field Pea (*Pisum sativum* L.)

**DOI:** 10.3389/fpls.2019.00825

**Published:** 2019-07-10

**Authors:** Arpita Das, Ashok K. Parihar, Deepa Saxena, Deepak Singh, K. D. Singha, K. P. S. Kushwaha, Ramesh Chand, R. S. Bal, Subhash Chandra, Sanjeev Gupta

**Affiliations:** ^1^Bidhan Chandra Krishi Viswavidyalaya, Mohanpur, India; ^2^ICAR – Indian Institute of Pulses Research, Kanpur, India; ^3^Chandra Shekhar Azad University of Agriculture and Technology, Kanpur, India; ^4^ICAR – Indian Agricultural Statistics Research Institute, New Delhi, India; ^5^Regional Agricultural Research Station, Assam Agricultural University, Jorhat, India; ^6^G. B. Pant University of Agriculture and Technology, Pantnagar, India; ^7^Banaras Hindu University, Varanasi, India; ^8^Regional Research Centre, Punjab Agricultural University, Ludhiana, India; ^9^Narendra Deva University of Agriculture and Technology, Faizabad, India; ^10^All India Coordinated Research Project on MULLaRP, ICAR – Indian Institute of Pulses Research, Kanpur, India

**Keywords:** rust, GGE biplot, repeatability, desirability index, host plant resistance, field pea

## Abstract

Rust caused by *Uromyces viciae-fabae* is a major biotic constraint to field pea (*Pisum sativum* L.) cultivation worldwide. Deployment of host-pathogen interaction and resistant phenotype is a modest strategy for controlling this intricate disease. However, resistance against this pathogen is partial and influenced by environmental factors. Therefore, the magnitude of environmental and genotype-by-environment interaction was assessed to understand the dynamism of resistance and identification of durable resistant genotypes, as well as ideal testing locations for rust screening through multi-location and multi-year evaluation. Initial screening was conducted with 250 diverse genotypes at rust hot spots. A panel of 23 promising field pea genotypes extracted from initial evaluation was further assessed under inoculated conditions for rust disease for two consecutive years at six locations in India. Integration of GGE biplot analysis and multiple comparisons tests detected a higher proportion of variation in rust reaction due to environment (56.94%) as an interactive factor followed by genotype × environment interaction (35.02%), which justified the requisite of multi-year, and multi-location testing. Environmental component for disease reaction and dominance of cross over interaction (COI) were asserted by the inconsistent and non-repeatable genotypic response. The present study effectively allocated the testing locations into various categories considering their “repeatability” and “desirability index” over the years along with “discrimination power” and “representativeness.” “Mega environment” identification helped in restructuring the ecological zonation and location of specific breeding. Detection of non-redundant testing locations would expedite optimal resource utilization in future. The computation of the confidence limit (CL) at 95% level through bootstrapping strengthened the accuracy of the GGE biplot and legitimated the precision of genotypes recommendation. Genotype, IPF-2014-16, KPMR-936 and IPF-2014-13 identified as “ideal” genotypes, which can be recommended for release and exploited in a resistance breeding program for the region confronting field pea rust.

## Introduction

Field pea or dry pea (*Pisum sativum* L.) is widely cultivated on a global basis in West Europe, North America, India, Australia, Pakistan and South America, as a cool season food legume crop for human dietary protein and livestock (Kocer and Albayrak, 2012; Saxesena et al., 2013). It is predominantly an export-oriented cash crop of the world, constituting about 40 percent of the total trading in pulses (FAOSTAT, 2017). This crop is valued primarily due to richness in digestible proteins (21.2–32.9%), coupled with important minerals and vitamins, and thus, holds immense promise for alleviating protein malnutrition to the resource poor vulnerable sections of the society (Ceyhan and Avci, 2005). Envisaging the importance of this legume, significant contributions have been made in the recent past regarding genetic improvement and cultivar development. Unfortunately, biotic stresses *viz*. rust, powdery mildew, downy mildew, Ascochyta blight, and root rot are the major impedes in field pea cultivation, which have resulted in subsequent yield and biomass losses worldwide.

**FIGURE 1:**
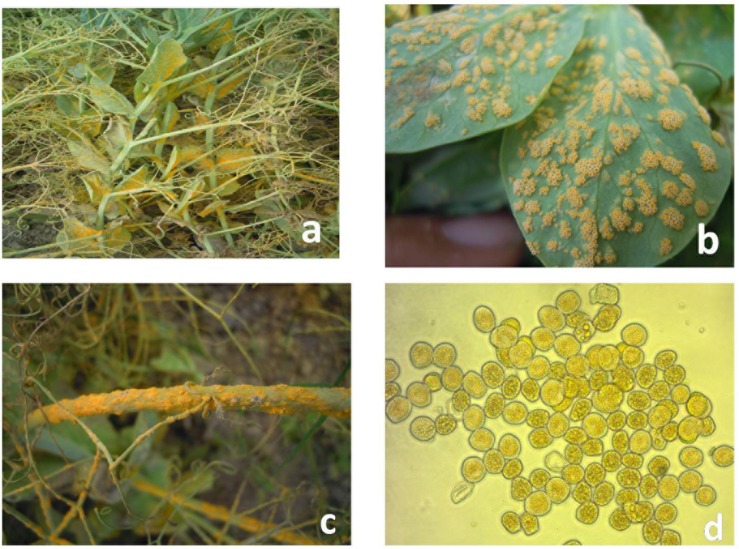
Symptoms of rust on infected field pea plants. **(a)** Infected field pea plants. **(b)** Infected field pea leaves. **(c)** Infected field pea stems. **(d)** Aeciospores of *Uromyces viciae-fabae.*

Field pea rust incited by *Uromyces* spp. currently has become a major concern in Europe, North and South America, India, China, Australia, and New Zealand (EPPO, 2012). The *Uromyces viciae-fabae* (Pers de Bary) is the causal organism for pea rust in tropical and subtropical regions *viz*. India and China (Xue and Warkentin, 2001; Vijayalakshmi et al., 2005; Kushwaha et al., 2006; Joshi and Tripathi, 2012; Singh et al., 2015). Reports of *U. pisi* (Pers.) (Wint.) causing fieldpea rust in temperate regions of Spain, Canada, and Egypt are also available in literature (Emeran et al., 2005; Barilli et al., 2009a, 2009b). However, *U. viciae-fabae* is autoecious and cosmopolitan in nature and attacks all aerial parts of the plant (Figure 1). The pathogen mainly appears during mid-spring at the reproductive stage of the crop, starting from flowering initiation to pod development, which resulted in reduction of photosynthetic area with an underdeveloped pod on affected plants, along with yield losses ranges from 57–100% (Upadhyay and Singh, 1994). Occurrence of the disease at early growth stages may result in complete failure of the crop. Thus, management of rust is a vital endeavor for sustainable field pea production. Chemical control is not holistic approach for controlling pea rust due to complexity in pathogen behavior. Wider host range, lack of durability in resistance of this airborne pathogen and quantitative nature of pea rust resistance are the crucial factors complicating disease management (Barilli et al., 2009a). Therefore, exploitation of host pathogen resistance is the most modest approach of rust control (Rubiales et al., 2013).

In grain legumes – rust pathosystems, mostly incomplete resistance with no host cell necrosis is reported (Sillero et al., 2006). In some legumes, hypersensitive reaction is also observed (Stavely et al., 1989; Sillero et al., 2000). However, in field pea, only incomplete resistance is observed against *U. viciae-fabae* (Xue and Warkentin, 2002; Chand et al., 2006) and *U*. *pisi* (Barilli et al., 2009c). The genetic basis of resistance to *U. viciae-fabae* is reported either under oligogenic (Katiyar and Ram, 1987) or polygenic control (Vijayalakshmi et al., 2005). Since there is existence of variants in both the host and the pathogen, understanding the host-by-pathogen interaction patterns for a particular host–pathogen system can be difficult and challenging (Yan and Falk, 2002). Thus, identification of stable and durable resistance genotypes of field pea against rust, followed by utilization of these genotypes as donors in a resistance breeding program would be a holistic attempt for disease management in a reliable way.

Understanding the role of environments and genotype by environment interaction (GEI), concerning the pathosystem and host genotype stability across diverse locations, is imperative for an efficient resistance breeding program. Environmental influence toward host pathogen response often deludes identification and recommendation of genotypes with durable resistance, thus, it is vital to identify “hot spots” having “repeatability” for evaluating genotypes and assessing actual value concerning respective disease. Unfortunately, reports are quite meager concerning appraisal of field pea genotypes against durable rust resistance across different environments, which creates exigency to understand the dynamics of host genotype and pathosystem under varied locations. Various stability approaches have been widely used in recent years to determine the GEI interaction regarding disease resistance through multi-location trials (MLT) in different crops (Abamu et al., 1998; Robinson and Jalli, 1999; Forbes et al., 2005; Mukherjee et al., 2013; Tekalign et al., 2017). Among these, GGE biplot methodology, which is a graphical approach, is becoming increasingly popular among the researchers for better explication of genotype and environmental evaluation. Recently, GGE biplot has been deployed to appraise genotypes with wide or specific adaptation related with resistance to different pathogens *viz*. in faba bean for Ascochyta blight and chocolate spot (Rubiales et al., 2012; Tekalign et al., 2017), in chickpea for fusarium wilt and ascochyta blight (Sharma et al., 2012; Pande et al., 2013), in pigeonpea against sterility mosaic disease (Sharma et al., 2015), in lentil for fusarium wilt and rust (Parihar et al., 2017a, 2018) and in mungbean against MYMV (Alam et al., 2014; Parihar et al., 2017b). Although, in the previous studies, during the assessment of test locations, “repeatability” and “desirability index” were not lucidly addressed for proper delineation of “mega environment.” Moreover, in the previous reports, genotypes and environments recommendation was based on only graphical biplot approaches without involving sound statistical assumptions, thus created perplexity toward the validity of the recommendations.

GGE biplots have not been expanded previously to appraise host genotypes response toward rust disease across varied locations, for identification of the best resistant genotypes, as well as “ideal” testing locations for better differentiation of resistance level among field pea genotypes. Hence, the present study was attempted through GGE biplot approach to enumerate the effect of GEI on field pea rust tested across various locations over the years, for identifying stable and superior field pea genotypes that could be recommended for future cultivation in the areas confronting rust problem. Additionally, the aim of the present study was to assess the influence of environments on host pathogen response along with identification of “ideal” test locations followed by grouping of various test locations into distinct “mega-environments” for optimum resource allocation in future testing. In the present study, integration of bootstrapping for generating confidence limit (CL) at the 95% level validated the genotypes recommendation.

## Materials and Methods

### Initial Testing

In a preliminary screening under the aegis of AICRP on MULLaRP, Kanpur, India (All India Co-Ordinated Research Project on Field pea and other pulses), a total of 250 genotypes of field pea, consisting of released varieties, germplasm accessions and advance breeding lines, were evaluated against rust reaction at nine locations during 2013–2014 in Augmented Block Design. Each genotype was sown in a plot of three rows of 3-meter length, spaced at 40 cm, and plant to plant distance was maintained at 10 cm. All the testing locations are decisively selected for the prevalence of *U. viciae-fabae*. Spreader rows of rust susceptible check were planted after every 10 rows of the test populations and five rows of each of the spreader row on all the sides of experimental area. A uniform basal dose of 20 kg: N, 40 kg: P_2_O_5_ and 40 kg” K_2_O was applied at the time of sowing. On such preliminary evaluation, a subset of 23 promising field pea genotypes based on their rust resistance reaction was extricated for multi-location and multi-year evaluation.

### Multi-Environment Evaluation (MEE)

**FIGURE 2:**
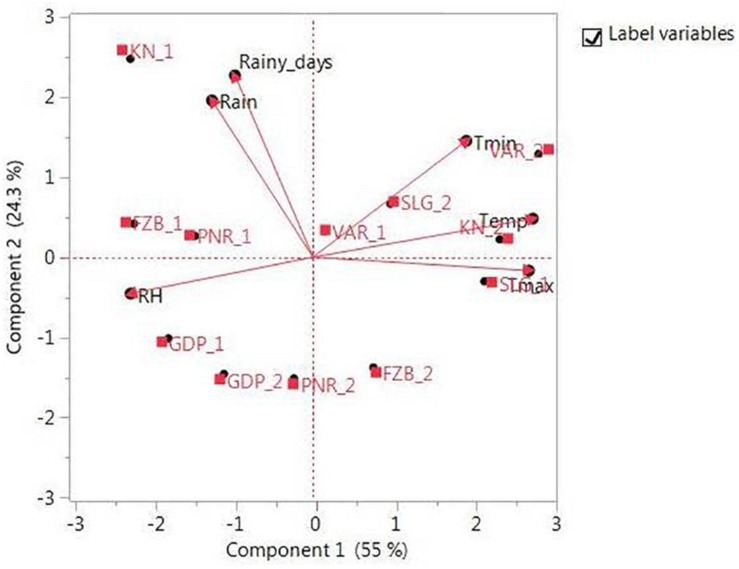
Principal component analysis (PCA) illustrating significant difference among test environments. Locations are: For Year-1 (2014--2015): FZB_1, Faizabad; GDP_1, Gurdaspur; KN_1, Kanpur; PNR_1, Pantnagar; SLG_1, Shillongani; and VAR_1, Varanasi. For Year-2 (2015--2016): FZB_2, Faizabad; GDP_2, Gurdaspur; KN_2, Kanpur; PNR_2, Pantnagar; SLG_2, Shillongani; and VAR_2, Varanasi.

The promising 23-field pea genotypes (Table 1) identified in preliminary screening were further evaluated for rust reaction across six diverse locations (Table 2) during winter season in two consecutive years (2014–2015 and 2015–2016) under natural epiphytotic condition. The aecial strain of *U. viciae-fabae* was present at all the testing locations. The genotypes were planted as per the standard agronomic practices following proper plant geometry with 4 m row length, 40 cm × 10 cm row to row and plant to plant spacing, respectively. A standard susceptible check “HFP 4” was sown after every 3 rows as spreader infector row for maintaining sufficient disease pressure under natural condition. Five rows of each of the spreader row were also grown around the experimental area. Potted spreader plants heavily infected with *U. viciae-fabae* were kept throughout the field to serve as additional sources of inoculumn. To increase the humidity, fields were irrigated at regular intervals until the grain attained full size. Further, to elucidate the difference among the test environments, principal component analysis (PCA) was performed considering various weather parameters: *viz*. max. and min. temperature, rain, rainy days and relative humidity of the locations (Figure 2). The results of PCA analysis validated the significant difference among the selected environments.

**TABLE 1:** Information regarding the field pea genotypes.

**Sl No.**	**Genotype**	**Pedigree**	**Developing center**	**Days to 50% flowering**	**Days to maturity**
1	HFP-4	T 163 × EC 190196	CCS Haryana Agricultural University, Hisar, India	68	113
2	Adarsh (IPF-99-25)	PDPD 8 × Pant P 5	ICAR-Indian Institute of Pulses Research, Kanpur, India	63	104
3	HFP-529	(HUDP 9 × Arkel) × (HUDP 12 × Arkel)	CCS Haryana Agricultural University, Hisar, India	66	120
4	HFP-715	DMR 50 × HFP 9948	CCS Haryana Agricultural University, Hisar, India	72	136
5	HFP-9907	Rachna × Bonnevilla	CCS Haryana Agricultural University, Hisar, India	69	121
6	HUDP-15	PG 3 (PG 3 × S 143) FC 1	Banaras Hindu University, Varanasi, India	76	125
7	IPF-2014-13	EC 538005 × IPFD 1-10	ICAR-Indian Institute of Pulses Research, Kanpur, India	67	122
8	IPF-2014-16	IPF 99-25 × Arkel	ICAR-Indian Institute of Pulses Research, Kanpur, India	61	102
9	IPFD-2014-11	IPFD 99-13 × P 1297 -97	ICAR-Indian Institute of Pulses Research, Kanpur, India	58	99
10	IPFD-2014-2	IPFD 99-13 × P 1297-97	ICAR-Indian Institute of Pulses Research, Kanpur, India	58	100
11	KPF-1023	HFP 9907B × EC 1	Agricultural Research Station, Kota, India		
12	KPMR-936	KPMR 65 × DDR 4	C.S. Ajad University of Agriculture and Technology, Kanpur, India	68	104
13	NDP-14-11	NDP 2 × HFP 4	N.D. University of Agriculture and Technology, Faizabad, India	68	122
14	Pant-P-243	Pant P 14 × Pant P 41	G.B. Pant University of Agriculture and Technology, Pantnagar, India	63	101
15	Pant-P-247	Pant P 25 × Pant p 66	G.B. Pant University of Agriculture and Technology, Pantnagar, India	62	103
16	Pant-P-250	Pant P 14 × Pant P 41	G.B. Pant University of Agriculture and Technology, Pantnagar, India	62	101
17	Pant-P-266	Pant P 86 × Pant P 13	G.B. Pant University of Agriculture and Technology, Pantnagar, India	61	101
18	Pant-P-269	P 1594 × T 163	G.B. Pant University of Agriculture and Technology, Pantnagar, India	66	102
19	Prakash (IPFD-1-10)	PDPD 8 × HUDP 7	ICAR-Indian Institute of Pulses Research, Kanpur, India	65	103
20	Vikash (IPFD-99-13)	HFP 4 × LFP 80	ICAR-Indian Institute of Pulses Research, Kanpur, India	63	102
21	VL-60	(JVP 14 × DMR 11) × VL 42	ICAR-Vivekananda Parvatiya Krishi Anusandhan Shansthan, Almora, India	57	100
22	VL-61	DDR 23 × VL 1	ICAR-Vivekananda Parvatiya Krishi Anusandhan Shansthan, Almora, India	58	101
23	Local rust susceptible check	–	–	–	–

**TABLE 2:** Description regarding the test locations in India.

												**Temp (^∘^C)**			
												
**Sl No.**	**Location**	**Biplot code**		**Elevation (msl)**	**Latitude and longitude**	**Rainy days**		**Avg. rainfall (mm)**		**RH (%)**		**Min**		**Max**	
									
		**(14–15)**	**(15–16)**			**(14–15)**	**(15–16)**	**(14–15)**	**(15–16)**	**(14–15)**	**(15–16)**	**(14–15)**	**(15–16)**	**(14–15)**	**(15–16)**
1	N.D. University of Agriculture and Technology, Kumarganj, Faizabad, Uttar Pradesh	FZB_1	FZB_2	113.0	26^0^47’N & 82^0^12’E	16	3	131	58	71	61	11	12	25	29
2	Regional Research Station, Gurdaspur, Punjab	GDP_1	GDP_2	219.0	32^0^02’N & 75^0^25’E	20	10	290	66	79	77	9	10	21	22
3	C.S. Azad University of Agriculture and Technology, Kanpur, Uttar Pradesh	KN_1	KN_2	152.4	26^0^29’N & 80^0^15’E	64	1	248	0.2	68	57	14	17	25	28
4	G.B. Pant University of Agriculture and Technology, Pantnagar, Udham Singh Nagar, Uttarakhand	PNR_1	PNR_2	243.8	29^0^00’N & 79^0^30’E	10	2	187	24.5	72	65	11	11	24	27
5	Regional Agricultural Research Station, Shillongani, AAU, Jorhat, Assam	SLG_1	SLG_2	50.2	26^0^00’N & 90^0^45’E	23	25	168	287	72	73	15	15	27	26
6	Banaras Hindu University, Varanasi, Uttar Pradesh	VAR_1	VAR_2	81.0	25^0^37’N & 82^0^97’E	20	24	106	42	74	30	13	16	28	32
															

### Disease Screening and Data Recording in MEE

The disease was assessed following the 1–9 scale of Subrahmanyam et al. (1995) described earlier. On the basis of disease scoring, the tested genotypes were classified into five distinct groups: (1) highly resistant; (2–3) resistant; (4–5) moderately resistant/susceptible; (6–7) susceptible; and (8–9) highly susceptible. Observation regarding rust was also recorded by visual estimation of leaf area covered with rust pustules (%).

### Construction of GGE Biplot

The GGE biplot was constructed based on the first two principal components (PCs) resulting from singular value decomposition (SVD), by estimating each element of the matrix through following formula (Yan et al., 2000; Yan and Kang, 2003):

$$Y_{\mathrm{ij}}=\mu +e_{j}+\sum_{n=1}^{N}\lambda_{n}\,\gamma_{\mathrm{in}}\,\delta_{\mathrm{jn}}+\varepsilon_{\mathrm{ij}}$$

Where,

Y_ij_ = mean response of ith genotype (i = 1,…,I) in the jth environment (j = 1,..,J).
μ = grand mean.
e_j_ = environment deviations from the grand mean.
λ_n_ = the eigen value of PC analysis axis.
γ_in_ and δ_jn_ = genotype and environment PCs scores for axis n.
N = number of PCs retained in the model.
ε_ij_ = residual effect∼ N (0,σ^2^).

For genotype evaluation, as well as determining stability, an “average environment coordination” (AEC) view of the GGE biplot has been constructed, which facilitates genotype comparisons based on mean of disease score and stability across environments within a “mega-environment” (Yan, 2001, 2002). A performance line passing through the origin of the biplot was used to determine the mean performance of the genotype in terms of rust scoring. The arrow on the performance line represents a decrease in stability of the genotype, i.e., higher susceptibility (Yan and Falk, 2002). Similarly, for evaluation of test environments, the “discriminating power vs. representativeness” view of the GGE biplot was constructed where the “ideal” test environment should be both discriminating of the genotypes and representative of the “mega-environment” (Yan et al., 2007). The “repeatability” of a test location was measured by the mean value of the genetic correlations between years within the location (Yan et al., 2011) for sustaining up consistency in genotypic performance. Additionally, a “desirability index” of the test locations has been enumerated, considering the association among the test environments and distance from the ideal genotype, based on the AEC, considering genotypic stability and adaptability (Yan and Holland, 2010). Regarding determination of relationship between test locations, angles between the various environment vectors were used to judge the correlation between the environments (Yan and Kang, 2003). Additionally, to ascertain superiority of the genotypes in different test environments, as well as grouping of test environments into different “mega environments,” a “which-won-where” view of the GGE biplot has been prepared (Yan and Rajcan, 2002). Finally, for assessing the validity of GGE biplot, bootstrapping, a nonparametric resampling approach, was deployed for construction of CL at the 95% level for individual principal component scores of both genotypes and environments, as suggested by Yang et al., 2009. In the raw data, columns represented environments (*p* = 12) and rows represented genotypes (*n* = 23). Accordingly, the raw data was average-centered for each environment so that each of the *p* dimensions of raw data has a mean of zero. The row-wise non-parametric resampling was done from the data matrix to obtain the bootstrap samples. The number of bootstrap samples were chosen to be 40 times to the number of rows (*B* = 920). The endpoints of CLs at 95% were estimated for genotypic and environmental scores.

### Data Analysis

The effects of environments, genotype and their interactions were determined by analysis of variance (ANOVA) for across the locations and for each individual location, using mixed-model analysis in GENSTAT (trial version 18; VSN International, Hemel Hempstead, United Kingdom). The ANOVA explained the partition of variations due to the effect of genotypes, environment and their interaction. Mean significant difference within genotypes and environments was enumerated by LSD test at *P* = 0.05 probability level. An illustration of distribution pattern of rust score across genotypes and across environments was presented through box plot. Relatedness of the genotypes and environments was calculated using Ward method and represented through a hierarchical cluster. The GGE biplot analysis was done by using the R software (R Development Core Team, Vienna).

## Results

Field pea genotypes exhibited variable responses concerning rust reaction in the tested locations. The pooled ANOVA of rust reaction revealed that the effect of genotype, environment and the genotype x environment interactions were significant among the tested genotypes (Table 3). Relative contribution of each source of variation reflected that environment, and GEI contributed 56.94 and 35.02% of the total variation, respectively, which indicated the perplexing role of the environment toward rust reaction among the genotypes tested across the locations. Likewise, in the different testing locations, the effect of genotype, year and genotype x year interactions were significant toward rust reaction among the tested genotypes (Supplementary Table 1).

**TABLE 3:** Analysis of variance for rust incidence in 23 genotypes of field pea evaluated at six locations in India during Year-1 (2014–2015) and Year-2 (2015–2016).

**Sources of**	**Degree of**	**Mean sum**	***P* value**	**%**
** variation**	** freedom**	** of squares**		** contribution**
Environment (E)	11	103.5292^∗∗^	<0.001	56.94
Genotype (G)	22	6.715453^∗∗^	<0.001	7.39
Genotype × Environment (G × E)	242	2.894505^∗∗^	<0.001	35.02

*^∗∗^*P* < 0.01.*

**FIGURE 3:**
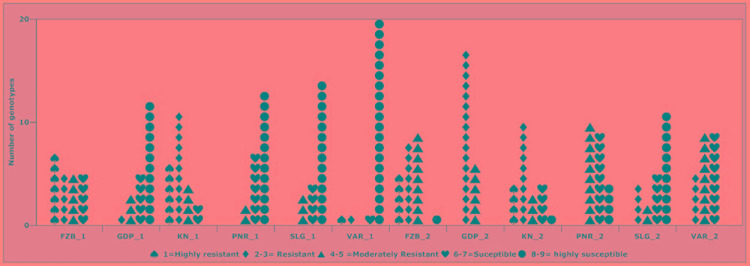
Frequency distribution of 23 field pea genotypes for rust assessment at six locations in India during Year-1 (2014–2015) and Year-2 (2015–2016). Locations are: For Year-1 (2014--2015): FZB_1, Faizabad; GDP_1, Gurdaspur; KN_1, Kanpur; PNR_1, Pantnagar; SLG_1, Shillongani; and VAR_1, Varanasi. For Year-2 (2015--2016): FZB_2, Faizabad; GDP_2, Gurdaspur; KN_2, Kanpur; PNR_2, Pantnagar; SLG_2, Shillongani; and VAR_2, Varanasi.

**FIGURE 4:**
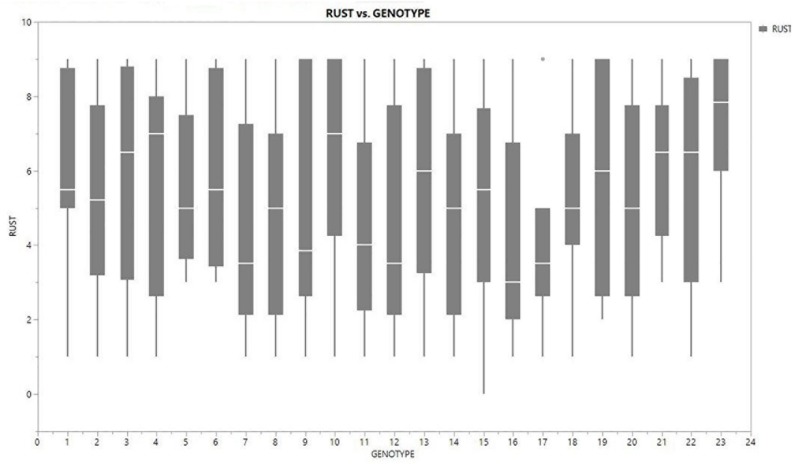
Boxplot view illustrating the distribution of rust assessment among 23 genotypes of field pea across six test locations. The box represents the area from the first quartile to the third quartile. A horizontal line goes through the box at the median. The whiskers (vertical line) go from each quartile to the minimum or maximum.

**FIGURE 5:**
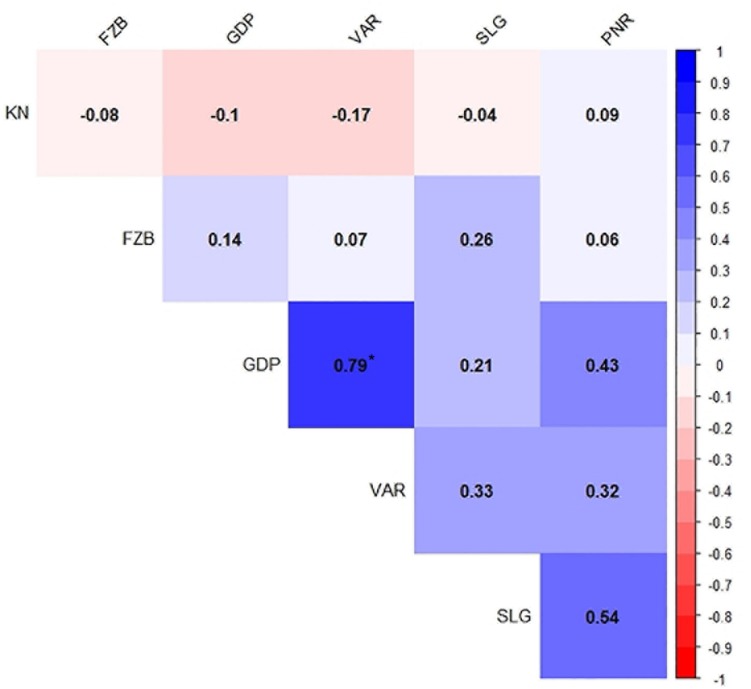
Spearman’s correlation between six test locations for field pea rust during Year – 1 (2014–2015) and Year – 2 (2015–2016). ^*^P < 0.05. Locations are: FZB, Faizabad; GDP, Gurdaspur; KN, Kanpur; PNR, Pantnagar; SLG, Shillongani; and VAR, Varanasi.

Inconsistent performance of the genotypes was observed over both the years and locations and elucidated through frequency distribution of rust reaction of the genotypes at each location (Figure 3). The average rust score of susceptible check (HFP-4) varied from 6.0–9.0 in both years and over the locations, advocating adequate disease pressure on the tested genotypes (Table 4). The magnitude of rust in the field pea genotypes over both the years and across the environments was illustrated through box plot view (Figure 4). Genotypes exhibited incongruous performance and reflected the presence of cross over interaction (COI) across the locations over both years. Undoubtedly, the highest rust scale was found in susceptible check with a mean rust score of 7.2. Across the locations and over both the years, Pant-P-250, Pant-P-266, IPF-2014-13, KPF-1023, KPMR-936, and Pant-P-243 were identified as moderately resistant genotypes. The association between testing environments in terms of rust score was tested by Spearman’s correlation analysis (Figure 5). It was observed that Kanpur exhibited a negative association with all the locations except Pantnagar, whereas rest of the five locations recorded a positive association with each other. The significant positive association between Gurdaspur and Varanasi confirmed that these locations have close resemblance regarding rust reaction among the tested genotypes.

**TABLE 4:** Mean rust scores of 23 genotypes of field pea at six locations during Year-1 (2014–2015) and Year-2 (2015–2016).

**Sl No.**	**Genotype**	**GDP**	**FZB**	**KN**	**SLG**	**PNR**	**VAR**	**Mean**	**LSD**
1	HFP-4	3.9	3.0	5.5	9.0	7.5	7.0	6.0	bdac
2	Adarsh (IPF-99-25)	4.6	3.0	2.0	8.5	6.5	7.0	5.3	ebdfhcg
3	HFP-529	6.1	2.0	4.0	8.6	6.5	8.0	5.9	ebdac
4	HFP-715	4.8	5.0	1.0	8.5	7.5	6.5	5.5	ebdfcg
5	HFP-9907	4.3	4.0	3.5	8.5	6.0	7.0	5.5	ebdfcg
6	HUDP-15	6.1	3.0	4.5	7.5	7.0	6.5	5.8	ebdfc
7	IPF-2014-13	5.8	2.0	2.0	4.9	6.0	6.0	4.4	fhg
8	IPF-2014-16	5.8	3.0	1.5	7.0	5.5	7.0	5.0	edfhcg
9	IPFD-2014-11	3.6	2.0	2.5	8.5	9.0	6.0	5.3	ebdfhcg
10	IPFD-2014-2	6.0	6.0	4.0	8.0	8.5	6.5	6.5	ba
11	KPF-1023	6.4	2.0	1.5	4.5	5.5	7.5	4.6	efhg
12	KPMR-936	4.8	2.0	2.5	5.5	7.0	6.5	4.7	edfhg
13	NDP-14-11	5.9	5.0	2.5	8.0	8.0	6.0	5.9	ebdac
14	Pant-P-243	4.8	4.0	5.5	4.5	5.0	4.5	4.7	edfhg
15	Pant-P-247	5.0	4.0	1.0	8.5	5.5	7.5	5.2	ebdfhcg
16	Pant-P-250	2.8	1.0	2.0	8.0	6.0	4.5	4.0	h
17	Pant-P-266	5.8	4.0	2.0	3.5	4.5	6.0	4.3	hg
18	Pant-P-269	6.3	6.0	3.5	6.0	4.5	6.5	5.5	ebdfcg
19	Prakash (IPFD-1-10)	5.8	4.0	2.0	8.5	7.0	8.0	5.9	ebdac
20	Vikash (IPFD-99-13)	3.8	4.0	2.5	9.0	7.5	3.5	5.0	edfhcg
21	VL-60	5.3	5.0	3.5	8.0	7.5	7.5	6.1	bac
22	VL-61	6.1	4.0	2.5	6.0	7.0	7.5	5.5	ebdfcg
23	Local Check	7.0	6.0	6.0	9.0	7.8	7.5	7.2	a
	Mean	5.2	3.7	2.9	7.3	6.6	6.5	5.4	

*LSD least Significant difference based t grouping. Mean value calculated by least Significant difference method.*

### Evaluation of Genotypes

**FIGURE 6:**
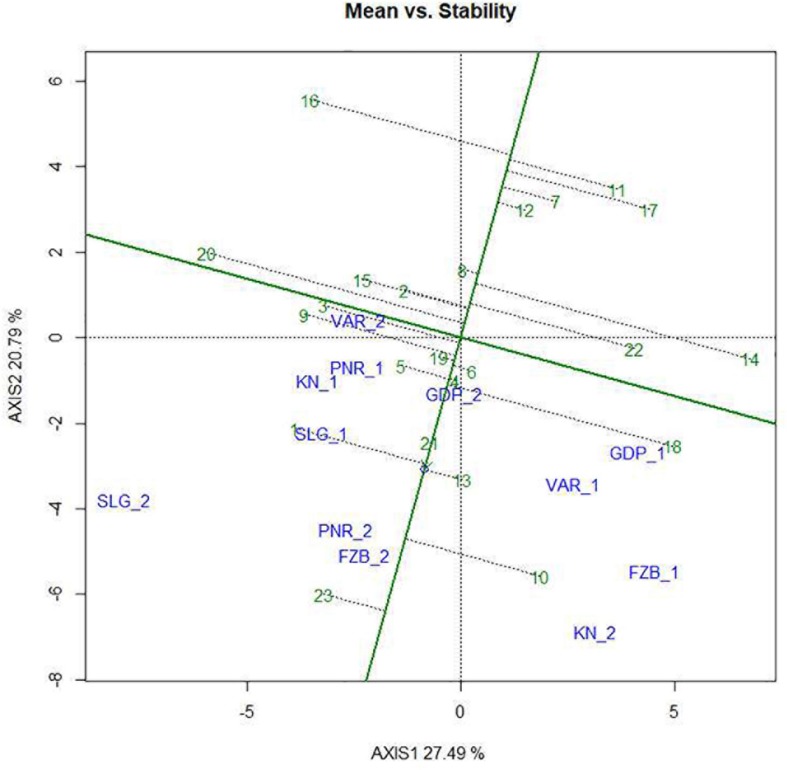
Mean vs. Stability view of the GGE biplot of 23 field pea genotypes across 6 testing locations. There was no transformation of data (transform = 0), and data were centered by means of the environments (centring = 2). The biplot was based on “row metric preserving.” Numbers correspond to genotypes as listed in Table 1. Locations are: For Year-1 (2014--2015): FZB_1, Faizabad; GDP_1, Gurdaspur; KN_1, Kanpur; PNR_1, Pantnagar; SLG_1, Shillongani; and VAR_1, Varanasi. For Year-2 (2015--2016): FZB_2, Faizabad; GDP_2, Gurdaspur; KN_2, Kanpur; PNR_2, Pantnagar; SLG_2, Shillongani; and VAR_2, Varanasi.

**FIGURE 7:**
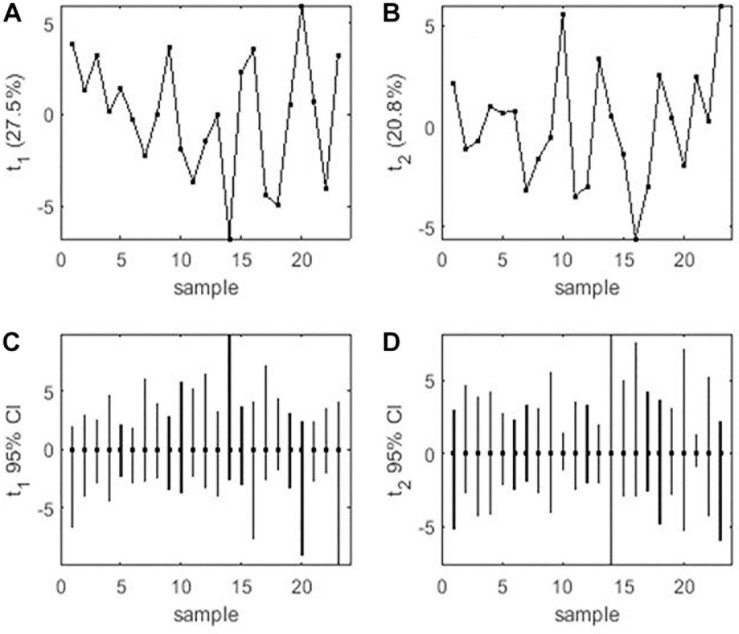
**(A)** PCA score values on PC1 vs. Genotype. **(B)** PCA score values on PC2 vs. Genotype. **(C)** PC1 score-values 95% BCa CLs (*B* = 920), shown centered on nominal score-values. **(D)** PC2 score-values 95% BCa CLs (*B* = 920), shown centered on nominal score-values. Numbers correspond to genotypes as listed in Table 1.

**FIGURE 8:**
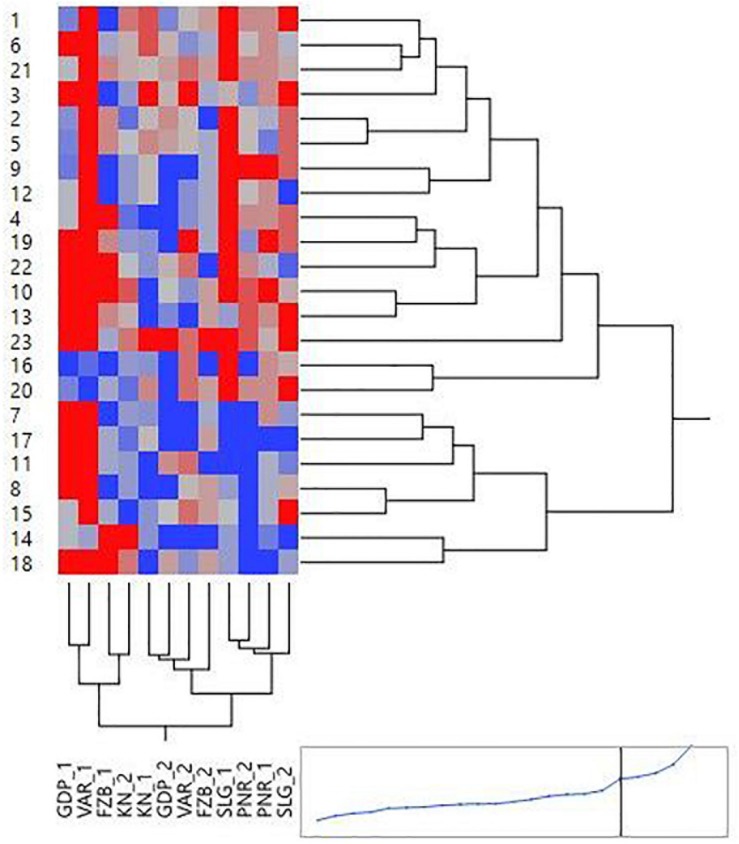
Hierarchical cluster analysis showing the relationship between 23 tested field pea genotypes against rust as well as 6 testing locations. Numbers correspond to genotypes as listed in Table 1. Locations are: For Year-1 (2014–2015): FZB_1, Faizabad; GDP_1, Gurdaspur; KN_1, Kanpur; PNR_1, Pantnagar; SLG_1, Shillongani; and VAR_1, Varanasi. For Year-2 (2015--2016): FZB_2, Faizabad; GDP_2, Gurdaspur; KN_2, Kanpur; PNR_2, Pantnagar; SLG_2, Shillongani; and VAR_2, Varanasi.

Mean performance and stability of the genotype across the locations were graphically portrayed through an “AEC” view of the biplot (Figure 6). The single arrow-head-line in the graph known as “AEC abscissa,” passing through biplot origin, indicates higher disease reaction. From the figure, it could be pointed out that Pant-P-250 (16), KPF-1023 (11), Pant-P-266 (17), IPF-2014-13 (7), KPMR-936 (12), and IPF-2014-16 (8) exhibited less rust reaction. Genotypic stability is generally assessed on the basis of the absolute length of the projection of a genotype. The best performing genotypes would be those with lowest disease reaction (higher negative projection on AEC) with highest stability, i.e., projection on AEC close to 0 (Yan, 2014). Accordingly, IPF-2014-16 (8) was the most “ideal” genotype, having short projection from “AEC abscissa” along with moderate resistance against rust. Genotypes located closer to the “ideal” genotype are more “desirable” than others. Therefore, KPMR-936 (12), followed by IPF-2014-13 (7), were considered as “desirable” genotypes, due to their closer position to the “ideal” genotype, with less rust score as well as having consistent performance. Considering the CL at 95% level concerning the individual genotypic and environmental scores corresponding to PC1 and PC2 (Supplementary Table 2), being enumerated through bootstrapping showed that the visible differences amid the genotypes reflected in the biplot were contributed to by the differences in the individual PC2 scores of the genotypes (Figure 7). It was also confirmed through CL at 95% level that the “ideal” genotype, IPF-2014-16 (8), was statistically different on the basis of PC2 scores (Lower limit: −3.60 and Upper limit: 0.67) from the two desirable genotypes, *viz*. KPMR-936 (12) and IPF-2014-13 (7). However, the two desirable genotypes were overlapping corresponding to their PC-2 scores and were not statistically different. Concerning rust reaction, all the tested field pea genotypes were grouped into three major clusters with 16 genotypes in cluster-I, five in cluster-II and only two in cluster-III (Figure 8).

### Evaluation of the Environments

**FIGURE 9:**
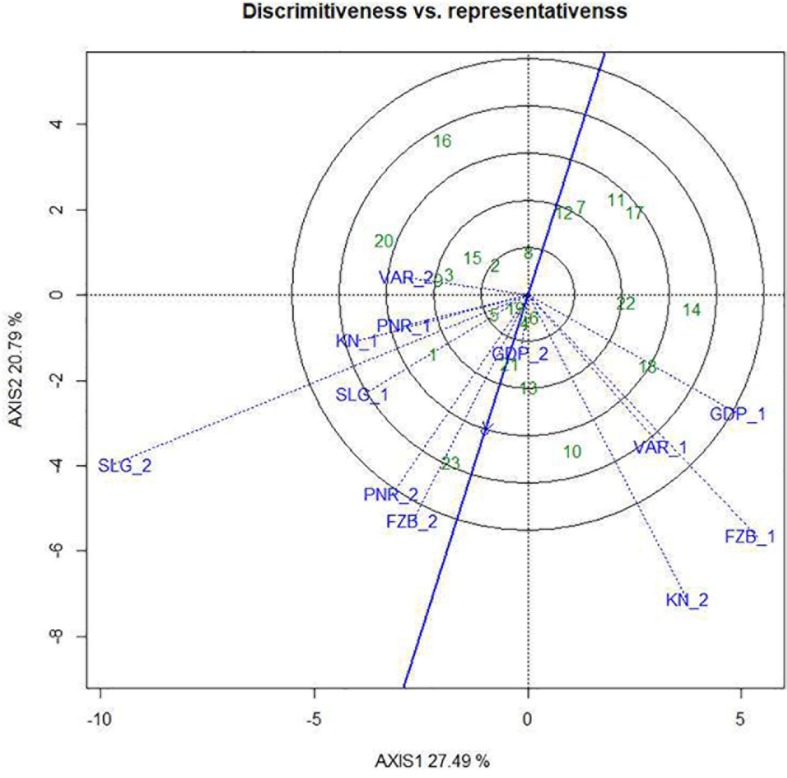
“Discrimitiveness vs. Representativeness” view of test locations based on GGE biplot of 23field pea genotypes across 6 testing locations. There was no transformation of data (transform = 0), and data were centered by means of the environments (centring = 2). The biplot was based on “row metric preserving.” Numbers correspond to genotypes as listed in Table 1. Locations are: For Year-1 (2014--2015): FZB_1, Faizabad; GDP_1, Gurdaspur; KN_1, Kanpur; PNR_1, Pantnagar; SLG_1, Shillongani; and VAR_1, Varanasi. For Year-2 (2015--2016): FZB_2, Faizabad; GDP_2, Gurdaspur; KN_2, Kanpur; PNR_2, Pantnagar; SLG_2, Shillongani; and VAR_2, Varanasi.

Among the test locations, during the first year, Faizabad exhibited longest environmental vector followed by Gurdaspur and Varanasi, whereas Pantnagar revealed shortest projection (Figure 9). Therefore, Faizabad was identified with most “discriminating locations” having the power of genotypes discrimination. On the contrary, during the second year (2015–2016), Shillongani exhibited longest vector with highest “discrimination” power followed by Kanpur and Faizabad. The single arrow-head-line in the graph is denoted as “AEC abscissa. The smaller angle between the environment vectors and “AEC abscissa” is the indicator of the locations having strong “representative” power. During the first year, Shillongani followed by Kanpur exhibited smallest angle with AEC, thus were identified as most “Representative” test locations, whereas, during the second year (2015–2016), Faizabad and Gurdaspur with high disease pressure were detected as being the most “representative” test locations. Although, Gurdaspur was recorded with lowest “discrimination” power in that year. Locations with high “discrimination” power with relatively less “representativeness,” such as *viz*. Faizabad and Pantnagar, should be considered for detecting stable genotypes. In the present study, over both years “repeatability” of the testing locations was assessed through visualizing their association ship. It was observed that amid all the locations over two years, Shillongani (*R*^2^ = 0.549), along with Pantnagar (*R*^2^ = 0.480), were revealed as highly “repeatable” locations, having the ability to exhibit consistent genotypic performance with non-cross over type of interaction (NCOI) toward rust invasion (Table 5). The “desirability index” of testing location is the overall manifestation of pooled performance based on the “discriminatory” power of a location and the “representativeness.” Based on two years of data, it could be concluded that Shillongani locations with highest “desirability index” were detected as “ideal” testing locations or “hot spots” for screening rust resistance in field pea genotypes (Table 5). Additionally, Faizabad, and Pantnagar would also be considered for field pea rust screening.

**TABLE 5:** Standardized test location evaluation parameters.

**Location**	**Discriminating**	**Represen-**	**Desirability**	**Repeatability**
	** power**	**tativeness**	** index**	***R*^2^ (%)**
GDP	4.514	0.429	1.453	22.4
FZB	7.053	0.480	3.066	28.6
KN	5.362	0.404	2.415	18.2
SLG	6.671	0.532	3.465	54.9
PNR	6.441	0.458	2.634	48.0
VAR	4.577	0.313	1.854	−17.8

### Identification of Mega Environments

**FIGURE 10:**
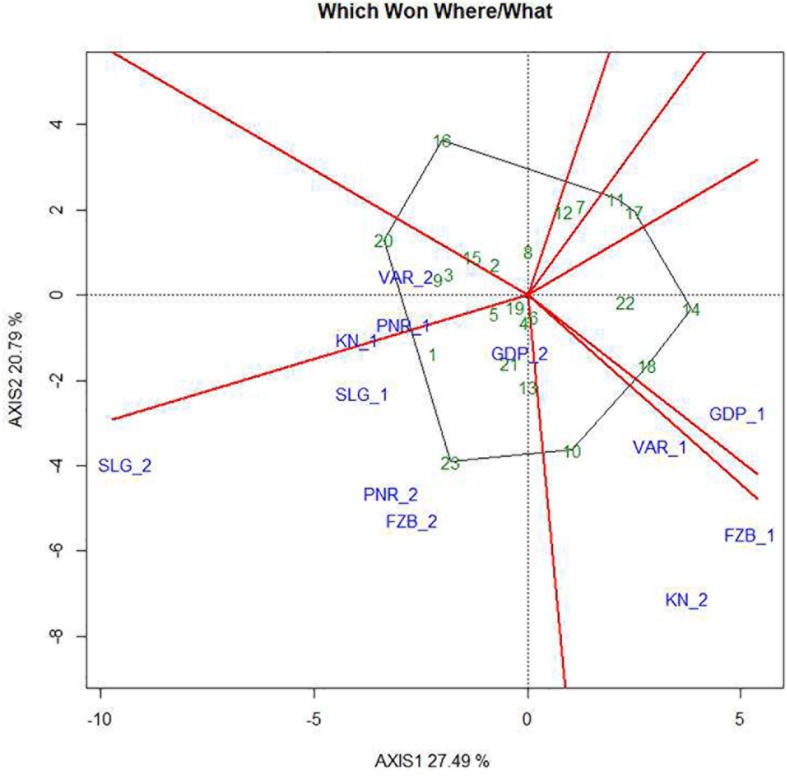
“Which-won-where” view of the GGE biplot of 23 field pea genotypes across 6 testing locations. There was no transformation of data (transform = 0), and data were centered by means of the environments (centring = 2). The biplot was based on “row metric preserving.” Numbers correspond to genotypes as listed in Table 1. Locations are: For Year-1 (2014--2015): FZB_1, Faizabad; GDP_1, Gurdaspur; KN_1, Kanpur; PNR_1, Pantnagar; SLG_1, Shillongani; and VAR_1, Varanasi. For Year-2 (2015--2016): FZB_2, Faizabad; GDP_2, Gurdaspur; KN_2, Kanpur; PNR_2, Pantnagar; SLG_2, Shillongani; and VAR_2, Varanasi.

The two-dimensional polygon view in the form of “which-won-where” polygon of GGE biplot is deployed to identify genotypes for a specific test environment. The perpendicular lines are drawn from the origin of the biplot to each side of the polygon for separating the biplot into several sectors, having one “wining” genotype for each sector located at the vertex of the polygon. In the present study, it was observed that Pant-P-250 (16) had the lowest rust susceptibility and was placed far from the origin depicting inconsistency in the performance (Figure 10). Additionally, Pant-P-266 (17), IPF-2014-13 (7), KPF-1023 (11), KPMR-936 (12), and Vikash (20) also exhibited low rust infection. Inversely, the local check (23) was located just opposite to Pant-P-250 (16), in the downstream from the origin, thus was revealed as the most susceptible genotype. Among all the genotypes revealing resistance to moderate resistance response, the most consistent performance was disclosed by IPF-2014-16 (8), which was placed adjacent to “AEC abscissa” with lowest projection onto the “AEC ordinate.” The equality lines partitioned the graph into four sectors during the first year, whereas in the second year, three sectors have been observed. These sectors could be entitled as “Mega Environment” affirming environmental variability and existence of COI. During the first year, Gurdaspur and Shillongani alone represented two different “mega environments” with distinct ecological features and genotypic responses toward rust. The other two “mega environments” were constituted by two locations in each, where Varanasi and Faizabad formed one “mega environment” and Kanpur and Pantnagar formed the other one. Deviation in the pattern of COI was reflected during the second year in contrast to the first year. In the second year, Kanpur and Varanasi alone constituted the two different “mega environments,” while the rest of the four locations formed the third one. Thus, considering rust response of the genotypes together, for both the years it was revealed that all the tested environments could be divided into four different “Mega environments.”

## Discussion

Fieldpea rust is gaining prominence in Europe, India and China as it causes huge yield losses. Management of rust becomes enigmatic due to wider host range of the pathogen along with quantitative nature of the host pathogen interaction. Moreover, the influence of weather variables obscures the scenario, which creates urgency of repeated appraisal of disease severity at diverse locations for searching out durable resistance sources. Environmental effect as well as complex GEI may reduce genetic gain under selection and further create a perplexing situation regarding selection and ranking of resistant genotypes. The presence of COI in different environments switches over the genotype ranking and reduces the correlation between phenotypic and genotypic values, thus advocating multi-environment screening of genotypes for drawing conclusions regarding genotypic superiority. Unfortunately, screening of foliar disease like rust is a kind of tedious and costly affair, particularly when natural screening is the only option where unpredictable weather parameters may change the disease spectrum (Sharma et al., 2016; Parihar et al., 2018). Multi-location testing creates a burden on resource poor states and, therefore, seeks attention for identification of “hot spot” or ideal testing locations as well as “mega environment” delineation considering multi-year data for disease resistance screening.

In the present study, GGE biplot (Yan and Kang, 2003) methodology was applied for assessment of rust resistance in field pea genotypes with general or specific adaptation beside appraisal of ideal test locations, and consequently discrimination of “mega environment” for restructuring of zonation. An attempt has also been made for precise recommendation of durable resistant genotypes against field pea rust through integrating bootstrapping for generating CL at 95%. Significant environment (56.94%) and GEI (35.02%) toward rust reaction was reflected in ANOVA (Table 3), and confirmed the impact of GEI and dynamic nature of rust disease spectrum in the tested environments. Testing locations with discrete agro-ecologies generated a differential response of the field pea genotypes and changed genotype ranking. Previous reports affirmed the role of environment and GEI, mystifying selection of stable genotypes with durable resistance against various pathogens (Pande et al., 2013; Alam et al., 2014; Sharma et al., 2015, 2016, Funga et al., 2017; Parihar et al., 2017a, 2017b, 2018).

The field pea genotypes had a significantly differential response toward rust under different testing locations, also validating GE influence. The rust reaction was relatively high in Shillongani followed by Pantnagar and lowest at Kanpur. In polycyclic disease like rust, inocula production is a crucial factor for determining the rate of epidemic and it is highly influenced by weather variables (Kushwaha et al., 2007). The tested genotypes in the present study also recorded variable responses in different locations, confirming the presence of COI, and thus implying the importance of multi-environment testing. Presence of COI is non-additive, non-separable in nature and suggesting for breeding of specific adaptation (Gregorius and Namkoong, 1986; Baker, 1990; Singh et al., 1999; Yan and Hunt, 2002; Rakshit et al., 2012; Xu et al., 2014). Differences in weather variables among the testing locations, as well as genetic variation in the host and pathosystem, ultimately generated variable genotypic response over the locations and over the years. Previous studies also stated incoherent genotypic responses with variable disease reaction in other crops (Alam et al., 2014; Sharma et al., 2015, 2016; Parihar et al., 2017a, 2017b). During screening, a sufficient disease score was corroborated by the consistent reaction of the susceptible check across the locations and over the years.

In the comprehensive plant breeding program, plant breeders prefer to delineate genotypes having the least interacting effect with environments with broad adaptation. Unfortunately, in resistance breeding program, this infrequently happens due to complexity between host pathogen interaction and consequence in disease prevalence. Multi-environment testing facilitates to find out genotypes having small spatial variable with consistent performance over locations, along with having small temporal variable with coherent performance over years (Kang, 2002). In the “Mean vs. Stability” view of the GGE biplot, the “AEC ordinates” signify higher GE interaction effect in both directions and represent poor stability (Yan and Tinker, 2006), whereas, the vector projections of the genotype to the “AEC abscissa” represent the average performance (Yan and Falk, 2002). In the present study, Pant-P-250 (16), KPF-1023 (11), Pant-P-266 (17), IPF-2014-13 (7), and KPMR-936 (12) exhibited higher negative projection on the ATC abscissa, thus less rust reaction. IPF-2014-16 (8) was identified as the most “stable” and “ideal” genotype with lowest projection onto the “AEC abscissa.” Additionally, in the present study, KPMR-936 (12) and IPF-2014-13 (7) were identified as “desirable” genotypes amid others and were positioned closer to the ideal genotype, IPF-2014-16 (8). Similarly to the “ideal” genotype, these two “desirable” genotypes also have the resistance response i.e., higher negative projection on the ATC abscissa with less projection on AEC ordinates i.e., high stability (Yan et al., 2007; Parihar et al., 2018). These strategies have been successfully deployed for identifying stable and resistant genotypes in different crops (Beyene et al., 2011; Sharma et al., 2015, 2017; Parihar et al., 2017a, 2017b; Sillero et al., 2017). Further, through deploying bootstrapping for enumeration of CL at 95%, it was confirmed that the ideal genotype, IPF-2014-16 (8), was statistically different from the two desirable genotypes, whereas, there was no statistical difference between the two desirable genotypes. Thus, the “ideal” genotypes, along with any one of the “desirable” genotypes with durable resistance, would be precious genetic resources in the future for the comprehensive resistance breeding program of field pea fronting rust issue. In the present study, integration of GGE biplot, along with a statistical hypothesis like bootstrapping, increased the precision of the visual observation toward genotypes recommendation.

During a multi-environment trial, plant breeders should meticulously screen out testing locations considering their “discrimination” power to categorize the genotypes, “representativeness” of the mega-environment of interest, “desirability index,” and “repeatability” across years in genotype ranking (Yan et al., 2011). Previous report stated that “representativeness” is the key factor to decide how a test location should be used in genotype evaluation, assuming adequate discriminating ability (Yan et al., 2007). Additionally, “repeatability” over the years and “desirability index” of the testing locations could be able to assess the “representativeness” of the testing locations flawlessly, allowing refinement in selection of future test locations. In the current study, during the first year, Faizabad and Shillongani appeared as the most “discriminating” as well as “representative” locations, respectively, while during the second year, the situation was reversed. Therefore, during the first year, Shillongani was identified as the “ideal” test location, and conversely during the second year, Faizabad was revealed as the “ideal” testing location. Dissimilar “ideal” environments in different years during the period of study were quite apparent and signified the highest contribution of environments among the total variation. During multi-environment testing, data from multi-year is essential for enumerating “repeatability” of the locations, for proper visualization of repeatability in genotype × environment interaction (Yan et al., 2000, 2007, 2011; Yan and Rajcan, 2002; Yan and Holland, 2010). Shillongani and Pantnagar, due to having consistent weather variables over both years regarding genotype response toward rust, were recorded as highly “repeatable” locations. Additionally, “desirability index” suggested that Shillongani followed by Faizabad were the “ideal” locations for rust screening. Finally, considering the four parameters (“discrimination,” “representativeness,” “repeatability,” and “desirability index”) in our study, all the testing locations have been classified in to four categories. Shillongani would be considered as “Type-I” or “ideal” testing locations, for screening out genotype at core location during early breeding stage.

Partitioning testing locations into distinct “mega environment” is the only way of getting consistent genotype performance within that particular sector. GGE biplot methodology can be successfully portrayed out “mega environment” through “which-won-where” view (Gauch and Zobel, 1997; Yan and Kang, 2003; Yan et al., 2007). The purpose of mega-environment identification is to understand the complex GEI pattern within that region for exploiting specific adaptation, as well as increment of selection responses (Yan et al., 2011). Previous reports defined “mega environment” consisting of locations exhibiting similar and repeatable genotypic responses across the years (Yan et al., 2000; Yan and Rajcan, 2002; Yan and Tinker, 2006). Conversely, “Non-repeatability” during “mega environment” selection in the present study was obvious due to non-repeatable association among the different locations, as well as inconsistency in genotypic and environmental scores (Krishnamurthy et al., 2017). Locations within each “mega environment” constructed in the present study revealed identical conclusions regarding genotypic response toward rust reaction. Judicial alignment of testing locations and converging breeding efforts in a location specific manner holds great relevance for improving the precision in the resistance breeding program.

The present study focussed on enlightening the influence of environmental and genotype- by- environment interactions, concerning the response of field pea genotypes toward rust. Incoherent response of the genotypes and locations across the years reflected the influence of environment toward volatility of rust score. Our study proficiently discriminated “ideal” and “desirable” genotypes for future rust screening of field pea in India. IPF-2014-16, KPMR-936 and IPF-2014-13 as “ideal” and “desirable” genotypes with consistent performance should be recommended for cultivation in the area fronting rust problem.

## Author Contributions

SG and KK designed the overall project. AD wrote the manuscript under the supervision of SG, RC, and AP. DS and AD analyzed the data. DSa, KS, KK, RB, and SC performed the phenotyping and disease scoring. SG, AP, and RC edited and finalized the manuscript.

## Conflict of Interest Statement

The authors declare that the research was conducted in the absence of any commercial or financial relationships that could be construed as a potential conflict of interest.
