# Supplementary material for: Deciphering Genotype-by- Environment Interaction for Targeting Test Environments and Rust Resistant Genotypes in Field Pea (Pisum sativum L.)
Source: Front Plant Sci. 2019 Jul 10;10:825. doi: 10.3389/fpls.2019.00825 (PMC6635599; doi:10.3389/fpls.2019.00825)
Supplement: Supplementary file 1 [file Table_1.doc]

**Supplementary Table 1:** Combined Analysis of variance across various locations for rust incidence in 23 genotypes of field pea evaluated at six locations in India Year-1 (2014-15) and Year-2 (2015-16)

| **Locations** | **DF** | **Sources of Variation** | | |
| --- | --- | --- | --- | --- |
|  |  | Year  (Y) | Geno  (G) | Geno x Year  (G x Y) |
| Faizabad (FZB) | 22 | 0.85 | 4.113 | 4.994 |
| Gurdaspur (GDP) | 22 | 225.768 | 2.263 | 1.308 |
| Kanpur (KN) | 22 | 1.761 | 4.105 | 1.118 |
| Pantnagar (PNR) | 22 | 30.838 | 2.981 | 0.964 |
| Shilongani (SLG) | 22 | 25.584 | 5.774 | 2.381 |
| Varanasi (VAR) | 22 | 135.674 | 2.587 | 3.583 |

* P<0.05, ** P<0.01

**Supplementary Table 2:** Deviations of mean rust score of 23 field pea genotypes tested at six environments over both the years from the corresponding environmental means. Genotype and environment scores corresponding to the PC1 and PC2 and at CLs at 95 % enumerated through bootstrapping

| Sl No. | **FZB_1** | **KN_1** | **SLG_1** | **PNR_1** | **VAR_1** | **GDP_2** | **FZB_2** | **KN_2** | **SLG_2** | **PNR_2** | **VAR_2** | **Mean** | **PC1** | **Lower limit** | **Upper limit** | **PC2** | **Lower limit** | **Upper limit** |
| --- | --- | --- | --- | --- | --- | --- | --- | --- | --- | --- | --- | --- | --- | --- | --- | --- | --- | --- |
| 1 | -2.69 | 2.26 | 0.95 | 0.54 | 0.74 | -0.26 | 1.39 | 2.87 | 2.44 | 1.17 | 0.17 | 5.98 | 3.87 | -0.21 | 5.52 | 2.11 | -2.29 | 5.11 |
| 2 | 1.31 | 0.26 | 0.95 | -0.46 | 0.74 | 0.74 | -2.61 | -2.13 | 1.44 | 0.17 | 0.17 | 5.27 | 1.30 | -1.56 | 4.11 | -1.11 | -3.28 | 3.53 |
| 3 | -2.69 | 3.26 | 0.12 | 0.54 | 0.74 | 0.24 | -0.61 | -1.13 | 2.44 | -0.83 | 2.17 | 5.87 | 3.21 | 0.99 | 5.54 | -0.75 | -4.66 | 2.46 |
| 4 | 3.31 | -1.74 | 0.95 | 0.54 | 0.74 | -0.51 | -0.61 | -2.13 | 1.44 | 1.17 | -0.83 | 5.54 | 0.13 | -3.35 | 4.14 | 1.00 | -2.51 | 5.10 |
| 5 | 1.31 | 1.26 | 0.95 | -1.46 | 0.74 | 0.49 | -0.61 | -0.13 | 1.44 | 0.17 | 0.17 | 5.54 | 1.38 | -0.24 | 3.28 | 0.65 | -1.04 | 3.71 |
| 6 | -0.69 | 2.26 | 0.95 | 0.54 | 0.74 | 0.24 | -0.61 | 0.87 | -0.56 | 0.17 | -0.83 | 5.77 | -0.26 | -2.53 | 1.29 | 0.78 | -1.35 | 2.44 |
| 7 | -2.69 | -0.74 | -3.05 | 0.54 | 0.74 | -0.51 | -0.61 | -1.13 | -1.79 | -1.83 | -1.83 | 4.44 | -2.24 | -4.73 | 2.05 | -3.20 | -5.39 | -1.24 |
| 8 | -2.69 | -1.74 | -1.05 | -0.46 | 0.74 | -0.51 | 1.39 | -1.13 | 0.44 | -1.83 | 0.17 | 4.96 | -0.06 | -2.14 | 3.87 | -1.59 | -3.60 | 0.67 |
| 9 | -2.69 | 0.26 | 0.95 | 1.54 | 0.74 | -0.51 | -0.61 | -1.13 | 1.44 | 3.17 | -1.83 | 5.27 | 3.66 | 1.47 | 6.16 | -0.55 | -4.21 | 3.83 |
| 10 | 3.31 | -1.74 | 0.95 | 1.54 | 0.74 | -0.01 | 1.39 | 3.87 | 0.44 | 2.17 | -0.83 | 6.50 | -1.85 | -4.90 | 2.88 | 5.57 | 4.79 | 6.97 |
| 11 | -0.71 | -1.74 | -3.05 | -0.46 | 0.74 | 0.74 | -2.61 | -1.13 | -2.56 | -1.83 | 1.17 | 4.56 | -3.66 | -5.60 | 0.09 | -3.47 | -5.66 | -1.56 |
| 12 | -2.69 | 0.26 | 0.95 | 0.54 | 0.74 | -0.51 | -0.61 | -1.13 | -4.56 | 0.17 | -0.83 | 4.71 | -1.49 | -3.92 | 3.57 | -2.99 | -5.38 | -0.72 |
| 13 | 1.31 | -1.74 | -1.05 | 0.54 | 0.74 | -0.26 | 1.39 | 0.87 | 2.44 | 2.17 | -1.83 | 5.90 | -0.03 | -3.94 | 2.46 | 3.31 | 1.94 | 4.77 |
| 14 | 3.31 | -0.74 | -1.05 | -1.46 | -2.26 | -0.51 | -2.61 | 5.87 | -4.56 | -1.83 | -1.83 | 4.71 | -6.77 | -9.25 | 1.99 | 0.49 | -5.61 | 8.05 |
| 15 | -0.69 | -0.74 | -0.13 | -0.46 | 0.74 | -0.01 | 1.39 | -3.13 | 2.44 | -1.83 | 1.17 | 5.24 | 2.31 | 0.00 | 5.57 | -1.37 | -4.21 | 2.76 |
| 16 | -2.69 | -0.74 | 0.95 | 0.54 | -5.26 | -0.51 | -2.61 | -1.13 | 0.44 | -1.83 | 1.17 | 4.04 | 3.53 | -2.29 | 7.55 | -5.57 | -8.33 | -0.83 |
| 17 | -0.69 | 0.26 | -3.05 | -2.46 | 0.74 | -0.51 | 1.39 | -2.13 | -4.56 | -1.83 | -1.83 | 4.29 | -4.40 | -6.43 | 1.97 | -3.00 | -6.18 | -0.26 |
| 18 | 3.31 | -1.74 | -1.05 | -2.46 | 0.74 | 0.49 | 1.39 | 2.87 | -1.56 | -1.83 | -0.83 | 5.46 | -4.95 | -6.76 | -2.40 | 2.52 | -1.24 | 5.68 |
| 19 | 1.31 | -0.74 | 0.95 | 1.54 | 0.74 | -0.51 | -0.61 | -1.13 | 1.44 | -0.83 | 2.17 | 5.88 | 0.53 | -2.34 | 2.98 | 0.45 | -2.18 | 3.47 |
| 20 | -0.69 | 1.26 | 0.95 | 0.54 | -7.26 | -0.51 | 1.39 | -2.13 | 2.44 | 1.17 | 1.17 | 5.04 | 5.94 | -1.57 | 7.98 | -1.98 | -7.09 | 3.85 |
| 21 | 1.31 | 0.26 | 0.95 | 0.54 | 0.74 | 0.49 | 1.39 | 0.87 | 0.44 | 1.17 | 1.17 | 6.13 | 0.72 | -1.74 | 2.58 | 2.45 | 1.69 | 3.77 |
| 22 | 3.31 | -0.74 | 0.95 | -0.46 | 0.74 | 0.24 | -2.61 | -0.13 | -3.56 | 1.17 | 1.17 | 5.52 | -4.06 | -6.02 | -1.66 | 0.25 | -3.75 | 4.56 |
| 23 | -0.69 | 3.26 | 0.95 | 0.20 | 0.74 | 1.99 | 5.39 | 2.87 | 2.44 | 2.17 | 1.17 | 7.22 | 3.19 | -6.54 | 6.83 | 6.00 | 2.33 | 8.04 |
